# A Patient With Focal Myositis and Primary Cutaneous Diffuse Large B-Cell Lymphoma: A Case Report

**DOI:** 10.3389/fonc.2021.658907

**Published:** 2021-03-23

**Authors:** Quanxin Wu, Cheng Xu, Li Wang

**Affiliations:** ^1^ Cadre Ward Two, General Hospital of Central Theater Command of the People’s Liberation Army, Wuhan, China; ^2^ Department of Oncology, General Hospital of Central Theater Command of the People’s Liberation Army, Wuhan, China

**Keywords:** statin, ezetimibe, focal myositis, PCDLBCL, CK-MB

## Abstract

We report a rare case of a 92-year-old male, with a history of statin/ezetimibe intake, complained of pain and swelling of left forearm. The patient was diagnosed with focal myositis at first. Symptoms aggravated after 2 months of immunomodulatory therapy, and accompanied with protrusion lesion at left elbow. Biopsy of the protrusion lesion turned out to be primary cutaneous diffuse large B-cell lymphoma.

## Introduction

Focal myositis (FM) is a rare and benign dysimmune disease characterized as a rapidly growing solitary mass within a single muscle, which usually involved in lower limbs and experienced self-regression in most cases (1). Although several reported cases suggest some triggering factors such as nerve lesions, traumatic muscle lesions and autoimmune diseases (2–4), the etiology of FM remains unclear.

Primary cutaneous diffuse large B-cell lymphoma (PCDLBCL), leg type is a rare malignancy, which make up approximately 4% of all cutaneous lymphomas and 20% of all primary cutaneous B cell lymphomas (CBCLs) (5). PCDLBCL commonly occurs on one or both legs, with 10 to 15% of cases involving other sites. The 5-year survival of PCDLBCL is approximately 50%, which is far worse than other CBCLs (6, 7).

Here we report an old male patient with left forearm lesion, considered FM at first, while diagnosed with PCDLBCL 4.5 months later, and the patient finally succumbed to severe infection.

## Case Presentation

A 92-year-old male, claimed left forearm swelling for 5 months, and aggravated with pain for 3 weeks. The patient denied recent special illness, travel and injury. Medical history included hypertension, sick sinus syndrome, cardiac pacemaker implantation and hyperlipemia. He had taken rosuvastatin 10 mg/ezetimibe 10 mg discontinuously for 20 months and withdrawn both for 5 months. Current medications are Amlodipine Besylate.

**Figure 1:**
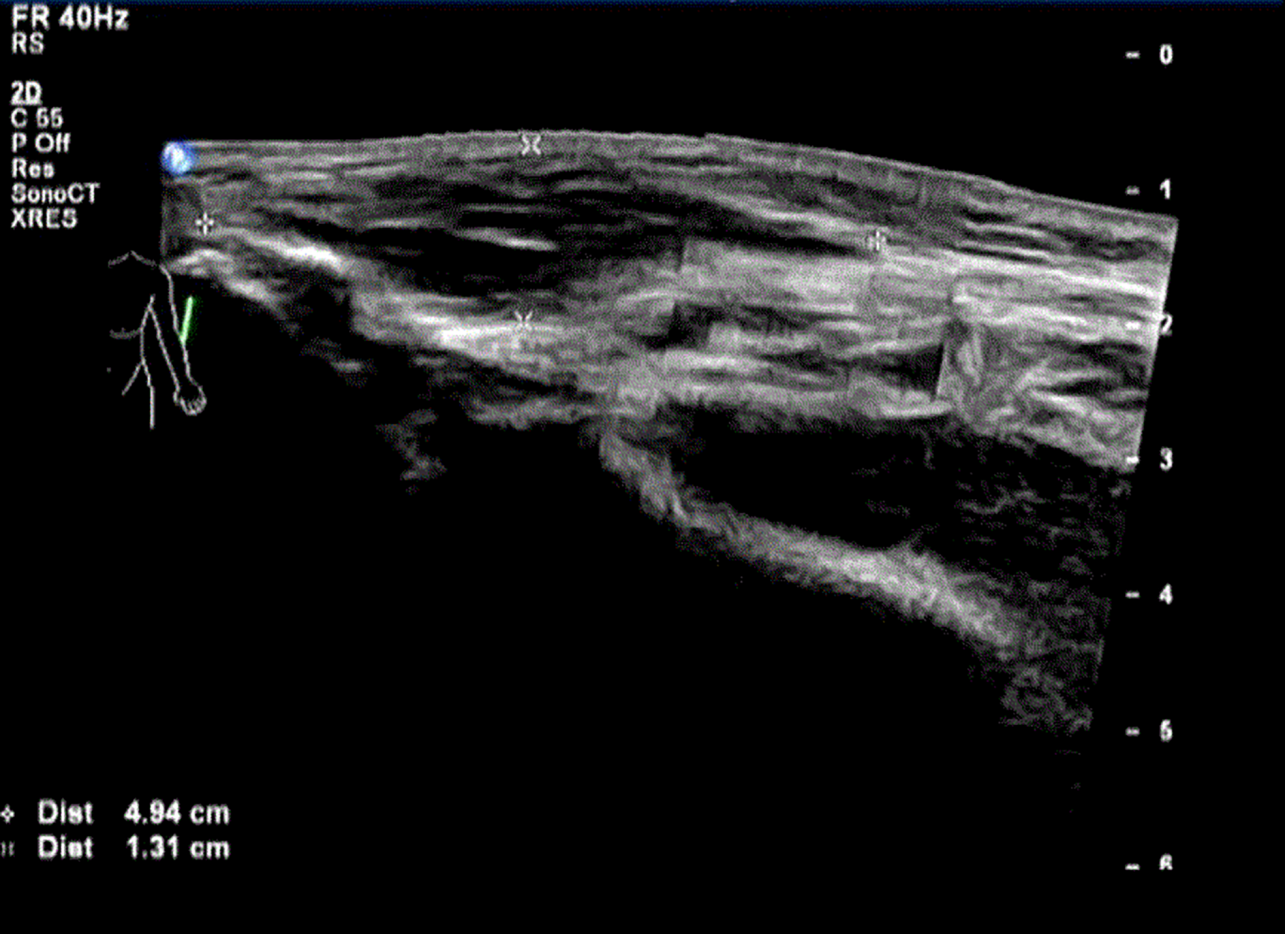
Ultrasonography of left forearm before the diagnosis of focal myositis.

**Figure 2:**
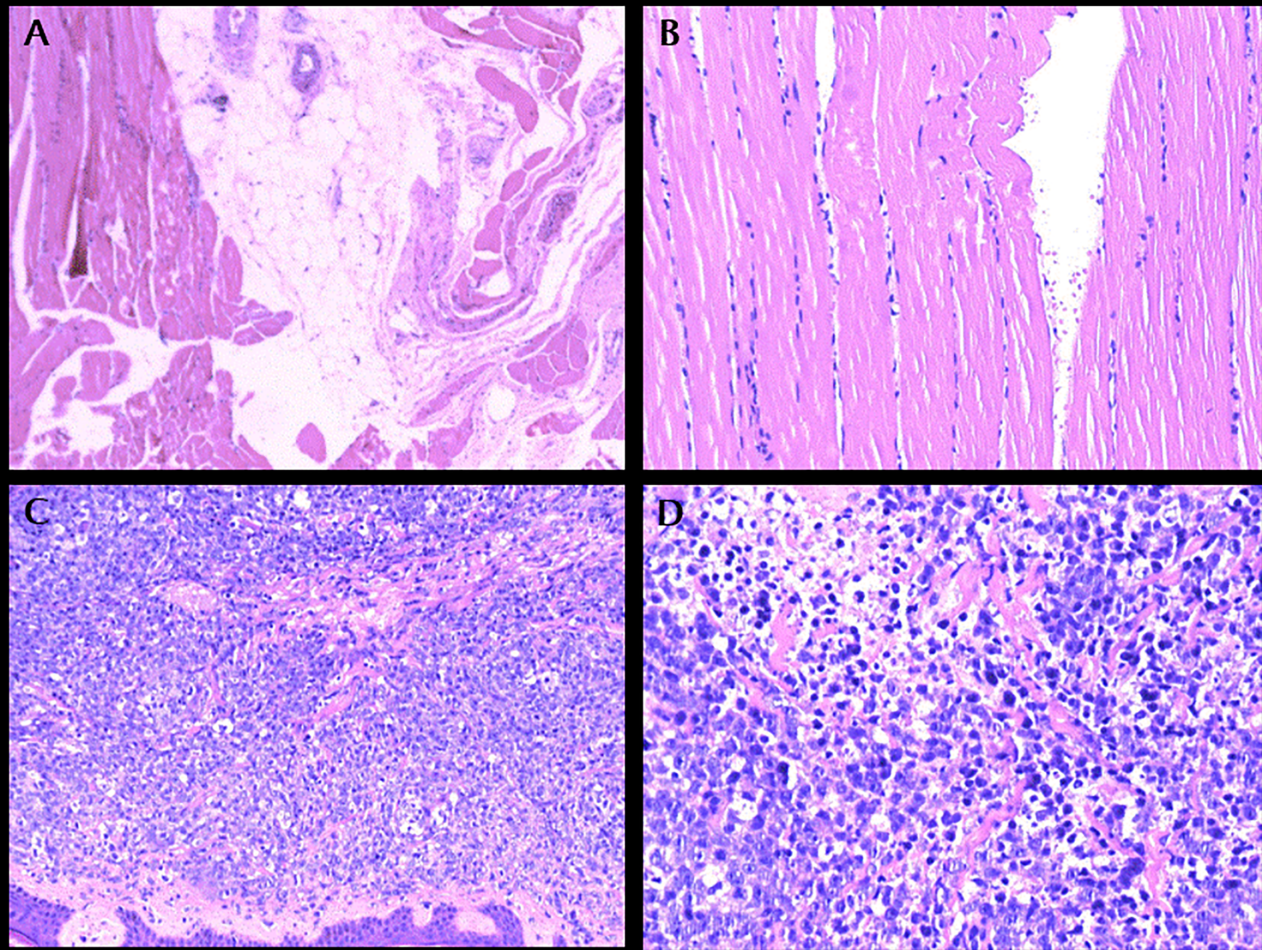
**(A)**: left forearm biopsy, HE × 100. **(B)**: left forearm biopsy, HE × 200. **(C)**: Protrusion lesion biopsy of left elbow, HE × 100. **(D)**: Protrusion lesion biopsy of left elbow, HE × 400.

Physical examination showed Lateral elbow joint swelling and obvious tenderness of left forearm. Laboratory test showed elevated level of creatine phosphate kinase isoenzyme (CK-MB 85.52 ng/ml), erythrocyte sedimentation rate (ESR), C-reactive protein (CRP) and Carcinoembryonic antigen (CEA) were within normal ranges. Antinuclear antibody assay showed positive result. The patient refused to accept magnetic resonance imaging, instead, Ultrasonography was performed and showed hypo-echoic area in superficial fascia layer of the left forearm (**Figure 1**). Ultrasonography guided incisional muscle biopsy were performed. Muscle biopsy (**Figures 2A, B** **)** showed focal muscle fiber degeneration, increased perimysial cells, and a few inflammatory cells infiltrate around the interstitial blood vessels.

**Figure 3:**
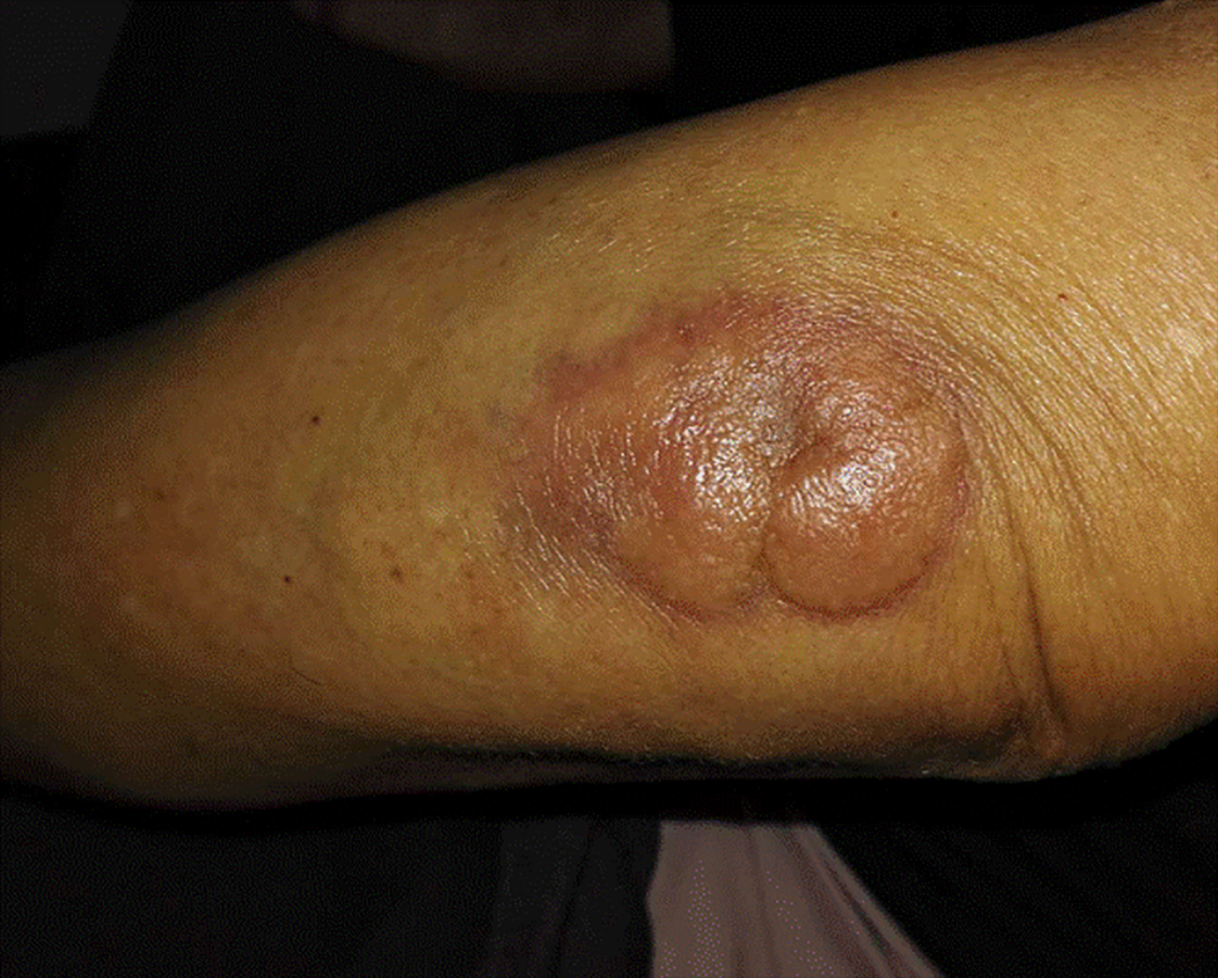
Protrusion lesion of left elbow.

**Figure 4:**
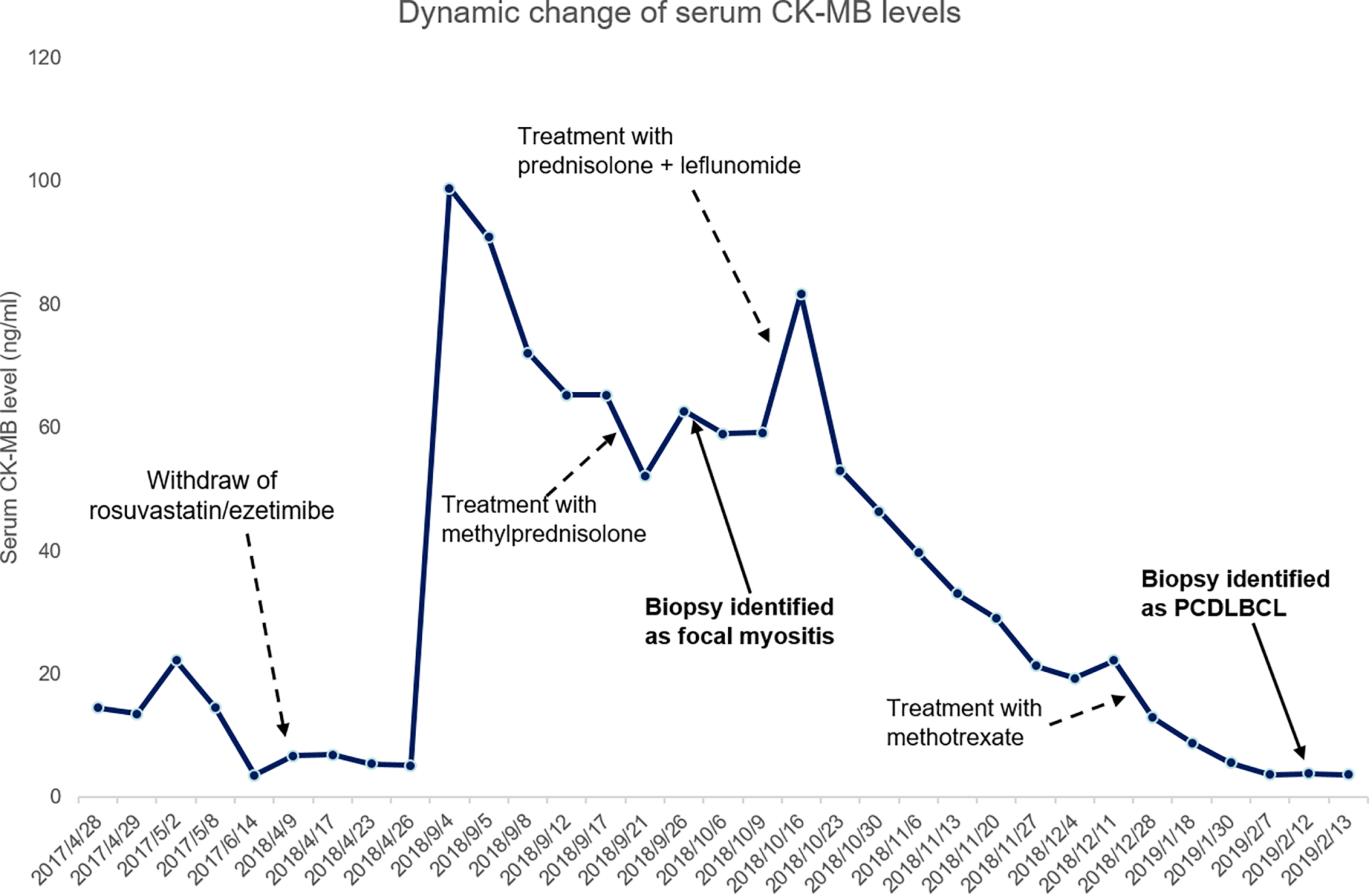
Dynamic change of serum CK-MB levels during the course.

Patient was diagnosed with focal myositis after a multidisciplinary consultation and treated with prednisone (initial dose 30 mg po qd, 5 mg dose reduction per week) and leflunomide (10 mg po qd). Left forearm swelling and tenderness improved accompanied with the decrease of CK-MB level. One month later, left forearm swelling aggravated. Leflunomide was replaced by methotrexate (10 mg po per week), and dosage of prednisone was increased to 30 mg po qd and kept for one month. The serum level of CK-MB decreased to normal and the dosage of prednisone was reduced to 25 mg po qd. But since then left forearm swelling was getting worse, accompanied with protrusion lesion at left elbow (**Figure 3**) and elevated skin temperature in local, meanwhile the patient claimed severe fatigue and lost self-care ability, regimen of methotrexate plus prednisone was abandoned. Another blood test showed an elevated serum CEA level (9.45 ng/ml), CRP level (174.15 mg/ml) and white blood cell count (11.9 ∗ 10^9^/L). Chest radiography indicated the existence of infection and Interstitial inflammation of both lungs. Protrusion lesion biopsy of left elbow was taken and antibiotics were used to control infection. The protrusion lesion biopsy turned out to be PCDLBCL (**Figures 2C, D** **)**, immunohistochemical staining: PCK (−), VIM (+), S-100 (−), HMB45 (−),CD3 (+, sporadic), CD20 (+, diffused), CD30 (−), Ki67 (+, >90%), EBER (−), ALK (−), CyclinD1 (−), CD5 (+, sporadic), CD15 (−), PAX-5 (+), CD79a (+), CD10 (−), CD21 (−), Bcl-6 (+), Mum-1 (+), P53 (+, sporadic), C-myc (+, 30%), Bcl-2 (+, diffused). Due to poor physical condition and severe lung infection, patient was suggested with best supportive care and died of respiratory failure. Dynamic change of serum CK-MB levels during the course were shown in **Figure 4**. Written informed consent was obtained from the patient at the diagnosis of PCDLBCL, ethical approval was waived according to the Declaration of Helsinki.

## Discussion

FM shared some clinical features with many diseases such as nodular or granulomatous myositis and soft tissue tumors, which brings difficulties for FM diagnosis. Clinical symptoms, magnetic resonance imaging (MRI), blood tests and biopsy help to make an accurate diagnosis. In our case, despite the lack of MRI, clinical course, physical examination, elevated CK-MB and pathology support the diagnosis.

In this case, the patient has received rosuvastatin/ezetimibe for a long time before the occurrence of left forearm tenderness. Statins are a group of drugs that reduce the levels of triglycerides and cholesterol in blood by inhibiting HMG-CoA reductase, an enzyme involved in rate limiting step in cholesterol synthesis, the incidence of statin-induced myotoxicity is estimated as 0.1–0.5% in cases of monotherapy (8). Ezetimibe inhibits cholesterol absorption from the small intestine by blocking the Niemann-Pick C1-like protein (9). Both statins and ezetimibe intake are reported to be associated with induction of myositis (10–12).

Interestingly, Ye et al. showed that statin intake may reduce the risk of diffuse large B cell lymphoma (13). Though statins may play a role in cancer prevention through cholesterol and cholesterol independent pathways (14–16), statins may induce myositis in rare situations, and inflammation is one of the cancer-causing factors (17, 18). In addition to our report, we noticed another reported case diagnosed with both statin-induced myopathy and extranodal marginal zone lymphoma (19). As we know, inflammation is one of the cancer-causing factor, in this case, the persistent focal myositis, possibly caused by statins/ezetimibe, may play a role in the oncogenesis of primary cutaneous large B-cell lymphoma.

## Data Availability Statement

The original contributions presented in the study are included in the article/supplementary material. Further inquiries can be directed to the corresponding authors.

## Ethics Statement

The studies involving human participants were reviewed and approved by the ethics committee of the General Hospital of Central Theater Command. The patients/participants provided their written informed consent to participate in this study. Written informed consent was obtained from the individual(s) for the publication of any potentially identifiable images or data included in this article.

## Author Contributions

QW offered the case and collected the data. CX prepared the manuscript. LW revised the manuscript. All authors contributed to the article and approved the submitted version.

## Conflict of Interest

The authors declare that the research was conducted in the absence of any commercial or financial relationships that could be construed as a potential conflict of interest.
